# Radiosensitizing high-Z metal nanoparticles for enhanced radiotherapy of glioblastoma multiforme

**DOI:** 10.1186/s12951-020-00684-5

**Published:** 2020-09-03

**Authors:** Jinyeong Choi, Gaeun Kim, Su Bin Cho, Hyung-Jun Im

**Affiliations:** Department of Applied Bioengineering, Graduate School of Convergence Science and Technology, Seoul National University, Seoul, Republic of Korea

**Keywords:** Radiotherapy, Gold nanoparticle, Glioblastoma, Radiosensitization, High-Z material

## Abstract

Radiotherapy is an essential step during the treatment of glioblastoma multiforme (GBM), one of the most lethal malignancies. The survival in patients with GBM was improved by the current standard of care for GBM established in 2005 but has stagnated since then. Since GBM is a radioresistant malignancy and the most of GBM recurrences occur in the radiotherapy field, increasing the effectiveness of radiotherapy using high-Z metal nanoparticles (NPs) has recently attracted attention. This review summarizes the progress in radiotherapy approaches for the current treatment of GBM, the physical and biological mechanisms of radiosensitization through high-Z metal NPs, and the results of studies on radiosensitization in the in vitro and in vivo GBM models using high-Z metal NPs to date.
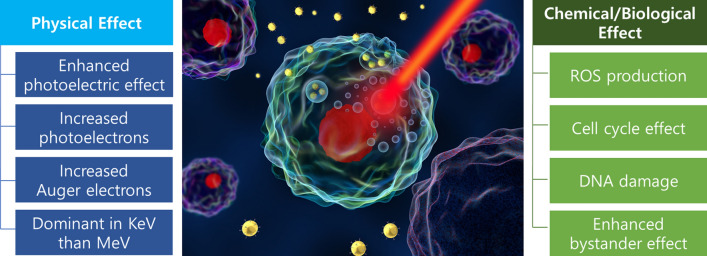

## Introduction

Glioblastoma multiform (GBM) is one of the most lethal malignancies with a 5-year survival rate of 5.5% and a median survival of 15 months [1]. Glioblastoma multiform is categorized as a grade IV astrocytic lineage glioma, and the most common brain malignancy, accounting for 47.1% of all malignant primary brain tumors, 82% of malignant gliomas, 56.1% of all gliomas, and 14.9% of all primary brain tumors [1, 2]. Glioblastoma multiform has an average annual incidence of approximately 11,000 in the United States. The incidence rate of GBM is higher in older people than in younger people and it is the highest in the age of 75–84 years. In addition, the incidence rate of GBM is 1.58 times higher in males than females [1]. The current standard of care for GBM includes surgery, temozolomide administration, and radiotherapy. Since GBM is considered as a radioresistant tumor [3] and most of the recurrence occurs in the radiotherapy field [4], radiosensitization of the tumor is an important target to improve the outcome in patients with GBM. Therefore, multiple radiosensitizing strategies are actively under development, including PI3K pathway inhibitors [5], DNA repair inhibitors [6], hyperthermia [7], aldehyde dehydrogenase inhibitors [8], and high atomic number (high-Z) metal nanoparticles (NPs) [9].

Nanomedicine has advantages over conventional cancer therapeutics such as multi-functionality, efficient drug delivery, and controlled release of the drug cargos [10, 11]. The efficient drug delivery in nanomedicine can be achieved either by passive targeting based on enhanced permeability and retention (EPR) effect or by active targeting after adding targeting moieties on the surface of the NPs [12, 13]. The controlled release strategies in nanomedicine include pH, thermal, and enzyme activated release [14]. Major drawbacks of nanomedicine compared to conventional small molecule-based therapeutics are as follows; 1) rapid elimination of the drug by the reticuloendothelial system (RES), 2) potential toxicity by inefficient excretion, and long-term retention in the body system [12, 15]. High-Z metal NPs are widely used in nanomedicine because of their unique abilities such as photothermal effect, fluorescence for optical imaging, photoacoustic effect, and radiosensitizing effects [16, 17]. Since high-Z metal NPs have a higher stopping power for ionizing radiation than soft tissue, they result in enhanced radiotherapy efficacy [18, 19]. Enhanced therapeutic effect by high-Z metal NPs mediated radiosensitization has been reported in multiple preclinical tumor models, including GBM [16]. Moreover, based on the success of preclinical studies, several clinical trials are underway to show the efficacy of the high-Z metal NP mediated radiosensitization. In particular, NBTXR3, a hafnium oxide NPs augmented radiotherapy, improved the pathological response in controlled phase 3 clinical trial in patients with soft tissue sarcoma [20].

In this review, we will focus on the potential of high-Z metal NPs application in the radiosensitization of GBM. First, we will describe the current standard of care for patients with GBM, with a focus on radiotherapy. Then, we will explain the mechanism of radiosensitization in physical and biological aspects, and summarize previous studies using high-Z metal NPs in radiotherapy for GBM. Finally, future perspectives of using high-Z metal NPs for radiosensitization in GBM will be presented.

## Radiotherapy for GBM treatment

**Fig. 1:**
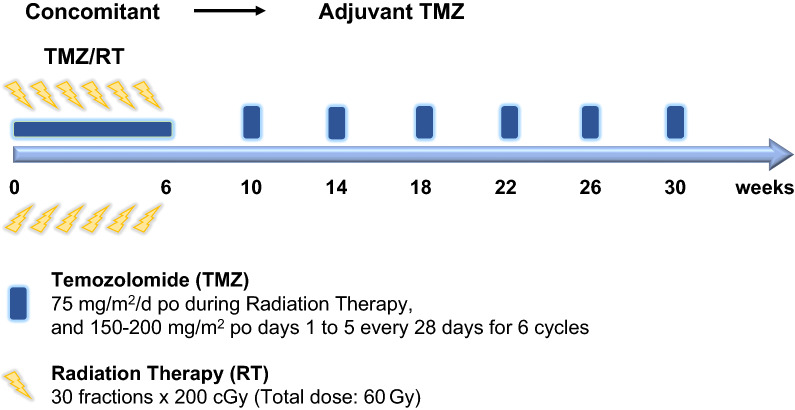
The schematic schedule of temozolomide treatment. This schedule included radiation therapy (RT) with treatment of temozolomide (TMZ). After the end of 6 weeks RT with TMZ of 75 mg/m^2^/d, the first of six adjuvant TMZ started. The 6 cycles of adjuvant TMZ was conducted with 150–200 mg/m^2^ po days 1 to 5 every 28 days (This figure was reconfigureated from the contents of Ref. [22])

Radiotherapy is one of the most effective and widely used cancer therapeutic modalities. About 50% of cancer patients are treated with radiotherapy either for curative, adjuvant, or palliative purposes [21]. The current standard of care for GBM includes maximal safe resection, concomitant daily temozolomide administration, and radiotherapy followed by adjuvant temozolomide treatment, which was established in 2005 (Fig. 1) [22]. The need for postoperative radiotherapy was strongly recommended due to the invasive nature of GBM, which makes complete resection with acceptable neurological results almost impossible. In the 1970s, several randomized clinical trials to demonstrate the benefit of radiotherapy were first performed by the Brain Tumor Study Group (BTSG). In the first clinical trial (BTSG 66-01), whole-brain radiotherapy (WBRT) resulted in prolonged survival (median survival: 8.4 vs. 3.5 months, P < 0.05) [23]. The second clinical trial (BTSG 69-01) also showed that the addition of WBRT resulted in improved survival compared to that in patients receiving only the best supportive care or chemotherapy (P = 0.001) [24].

Further retrospective studies were conducted to determine the optimal radiation doses. Based on the combined results of the previous randomized trials, median survival durations in patients who received less than 45 Gy were only 3–4 months; while those who treated with 50, 55, 60 Gy had a median survival of 8–9 months. The results indicated that WBRT higher than 50 Gy provided a better clinical outcome [25]. However further dose escalation beyond 60 Gy resulted in increased toxicity without significant survival benefit. Thus, current standard radiotherapy dose for GBM is determined as 60 Gy [26]. Until 1970, WBRT was advocated because of the initial assumption that GBM was a multicentric disease. However, this initial assumption was challenged by the finding that GBM recurred within 2 cm margin of the primary site in 90% of the cases [27]. Additionally, maximal dose of WBRT was limited by necrosis of the normal brain tissue and cognitive dysfunction [28, 29]. Therefore, the efficacy of involved-field radiotherapy (IFRT), defined as radiotherapy to the tumor and surrounding 3 cm geometric margins of the tumor, was suggested instead of WBRT. In a randomized trial, groups of patients who were treated with WBRT and IFRT were compared, and IFRT-treated patients had better survival than those who were treated with WBRT [30]. In addition, the standard fractionation of radiotherapy was reported to improve patient survival [25, 31]. However, further dose intensification methods such as hyperfractionation, accelerated fractionation, hypofractionation, hypofractionated boost, and/or stereotactic radiosurgery (SRS) boost did not show a convincing improvement in survival of general GBM patients, who are less than 70 years old with good performance score [32]. Therefore, 60 Gy of IFRT with standard fractionation (2 Gy/day) is the current standard radiotherapy for GBM [26].

Radiation therapy utilizes damaging DNA by direct or indirect ionization. That is, radiation can damage DNA using both physically direct ionization and using free radicals by water ionization. The goal of radiotherapy is to deliver the maximum dose to the target tumor tissue while sparing surrounding normal tissue [33]. Therefore, the maximum dose is determined by the toxicity to the surrounding healthy tissue. Recent advances in radiotherapy techniques such as intensity-modulated radiotherapy (IMRT), significantly improved dose conformity and clinical outcome by delivering multiple spatially modulated radiation fields [34]. To further improve radiotherapy efficacy, it is essential to develop drugs that can increase the efficiency of radiotherapy as well as to protect normal tissues [35]. Radioprotective drugs include free radical scavengers, cell cycle regulators, radiation-induced apoptosis inhibitors, and growth factors. Meanwhile, the radiosensitizers target epidermal growth factor receptor (EGFR), histone deacetylase, angiogenesis, DNA damage pathways, cell cycle regulators, cell death receptors, tumor hypoxia, and redox conditions. In particular, radiosensitizers are crucial for management of GBM because GBM is one of the most radioresistant cancers [36, 37]. In GBM, multiple clinical trials have been performed to test the radiosensitizing effect of EGFR inhibitors (erlotinib, everolimus), histone deacetylase inhibitors (valproate, vorinostat), antiangiogenic agents (vandetanib, enzastaurin), retinoic acid, glutamate inhibitor (talampanel), and a proteasome inhibitor (bortezomib). However, all clinical trials aimed to evaluate these radiosensitizers have been unsuccessful until now [38]. The cause of resistance to radiation therapy is due to various biological mechanisms and tumor heterogeneity. Mechanisms affecting radiation resistance include biological factors such as altered cell cycle, inflammation, altered DNA damage, hypoxic conditions, cancer stem cells, altered energy metabolism, and intertumoral and intratumoral heterogeneity [39]. More recently, high-Z metal NPs have been utilized to enhance the radiotherapy effect. Unlike the previous radiosensitizers, which target specific biological pathways, high-Z metal NPs primarily employ a strategy to enhance physical dose delivered during radiotherapy. It has been found that additional biological and chemical mechanisms are promoting the radiosensitizing effects of the high-Z metal NPs.

## High-Z metal nanoparticles for radiosensitization

The radiosensitizing effect by an iodine contrast agent was first demonstrated by Matsudaira et al. in 1980. The authors reported that the iodine contrast agent increased mammalian cells’ sensitivity to X-rays and caused chromosomal aberration in these cells [40]. Radiosensitization effect by high-Z metal was first observed in patients with metal implants who received radiotherapy for the treatment of mandibular [41] and head and neck cancers [42]. Thereafter, the radiosensitizing effect of high-Z metal NPs including gold, gadolinium, silver, bismuth, and different metal oxides NPs have been evaluated [16]. It was found that the radiation dose was enhanced when the radiation irradiated high-Z metal NPs because of the generation of secondary X-rays, photoelectrons, and Auger electrons [43]. The mechanism of radiosensitization by high-Z metal NPs will be further summarized in this chapter.

### Mechanism of radiosensitization: physical effect

Radiosensitization mechanism by high-Z metal nanoparticles can be explained by two different aspects, physical dose enhancement and subsequently increased biological reactions in the tissue [44]. The underlying rationale for physical dose enhancement is that high-Z metal has a higher stopping power of radiation than the soft tissue. While the Compton effect, photoelectric effect, and pair production occur when radiation is irradiated to the matter, high-Z metal NPs can induce higher energy deposition to the cancer tissue [45].

**Fig. 2:**
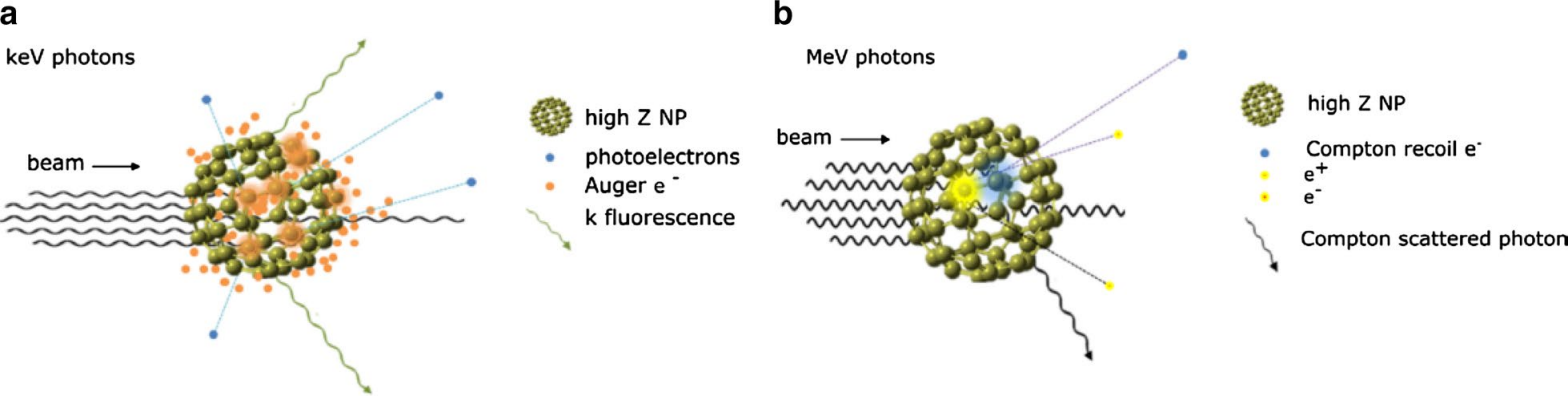
Schematic illustration of inelastic interactions with a high-Z nanoparticle for: **a** incident keV photons (orange clouds represent photoelectric events); **b** incident MeV photons (blue and yellow clouds represent Compton scatter and pair production events, respectively) (Reproduced with permission from reference: [47], copyright 2018 Institute of physics and engineering in medicine)

The Compton effect is the most crucial interaction between photons and tissue during the radiotherapy. In Compton scattering, photons collide with weakly bound electrons and give a portion of their energy to the electrons, and the electrons leave the orbit. At the same time, the incident photon is scattered after losing a portion of its energy. The photon continues to make additional interactions, and the electron begins to ionize the surrounding tissue (Fig. 2a). The probability of Compton interaction is inversely associated with the energy of the incoming photon. The Compton interaction is a dominating interaction in the photon energy range of 25 keV–25 MeV. As most radiation treatments are performed using energy levels of 6–20 MeV, Compton effect is the most common interaction in cancer tissue occurring during the radiotherapy. However, the probability of the Compton effect is independent of the atomic number of the material, so it is not substantially altered by the administered high-Z metal NPs [44, 45].

Pair production refers to an interaction between a photon and the nucleus of an atom. The photon gives its energy to the nucleus and creates a pair of a positively charged electron (positron) and an electron. The positron combines with the free electron and annihilates. The probability of pair production increases in proportion to Z^2^ and the energy of photon. Since the energy range where the pair production dominates is higher than 25 MeV, this interaction rarely occurs in routine radiation therapy.

The photoelectric effect mainly occurs when the material interacts with the relatively low energy ionizing radiation (< 60 keV). It is a phenomenon in which all the incident photon energy is absorbed by a tightly bound internal orbit electron and immediately deviates from its orbit. The incident photons disappear, and the repelled electrons are called photoelectrons. By this phenomenon, the outer orbital electron moves to the empty space of the internal orbit. It emits fluorescence photons at the wavelength that depends on the energy difference between the two orbits, which is also known as secondary radiation. After the photoelectric effect results in an inner shell vacancy, Auger electron can be emitted when an inner shell vacancy is filled by an outer shell electron [44]. The Auger electron has a very high linear energy transfer (LET), thus can be highly toxic to the cells [46]. The occurrence probability of the photoelectric effect increases sharply when the number of atoms (Z^3^) of the absorber increases, and dramatically decreases when the energy of the incident photons (E^3^) increases. That is, the photon mass attenuation coefficient is proportional to Z^3^/E^3^. The photoelectric effect has a relatively small contribution to the absorption in soft tissue; on the other hand, it is the dominant interaction in high-Z metal NPs. As a result, photoelectrons and secondary photons and Auger electrons emitted from high-Z metal NPs will cause highly localized dose enhancement and focal ionization of surrounding cells through photoelectric effects (Fig. 2b). Since the photoelectric effect tends to decrease with increasing photon energy, most pre-clinical studies on NPs and radiation therapy have used keV photons for optimizing the radiosensitization effect [47, 48]. It has been reported that high-Z metal NPs in the tumor could significantly increase the local dose, typically between 10 and 150 times more for kilovoltage photons. Taken together, physical dose enhancement by high-Z metal NPs is expected in KeV radiation because of the high probability of photoelectric effect, which is most significantly affected by high-Z metal NPs.

Monte Carlo methods have been used to evaluate the physical dose enhancement effect by high-Z metal NPs. In these methods, individual photon and electron interactions with matter are simulated probabilistically, based on measured cross-sections for different types of interactions. By modeling all of the interactions involving a given particle and any secondary particles, accurate predictions of the optimal dose can be made. The prediction takes into account factors such as beam attenuation, the distributions of secondary particles generated by interactions with soft tissue, and element variations in the irradiation field. A variety of dedicated packages have been developed to facilitate Monte Carlo simulations of radiation interactions with matter [49]. To prove the dose enhancement effect of NPs, Roeske et al. calculated the dose enhancement factor (DEF) in substances with atomic numbers from 25 to 90 by X-rays and brachytherapy sources such as ^125^I and ^103^Pd. As a result, it was confirmed that dose enhancement was found only in the elements having atomic numbers of 70 or more [50]. Also, Hossain et al. compared the dose enhancement of gold, platinum, and bismuth NPs by adjusting the size, concentration of NPs, and X-ray voltages. They calculated the production probability of photoelectrons, Auger electrons, and total DEFs according to the location of NPs in the endothelial cell and investigated how the type of element, concentration, location, size, and X-ray voltages were affecting the DEF. They found that maximum DEF could be achieved by bismuth element, small diameter of NPs, low energy of X-ray, and closer proximity of the NPs to the nucleus. Also, even though Auger electrons have lower energy than photoelectrons, Auger electrons have a more significant effect on dose improvement than photoelectrons. This is because Auger electrons have higher LET than photoelectrons; therefore, transfer higher energy to the cells adjacent to the NPs [51].

### Mechanism of radiosensitization: chemical/biological effect

**Fig. 3:**
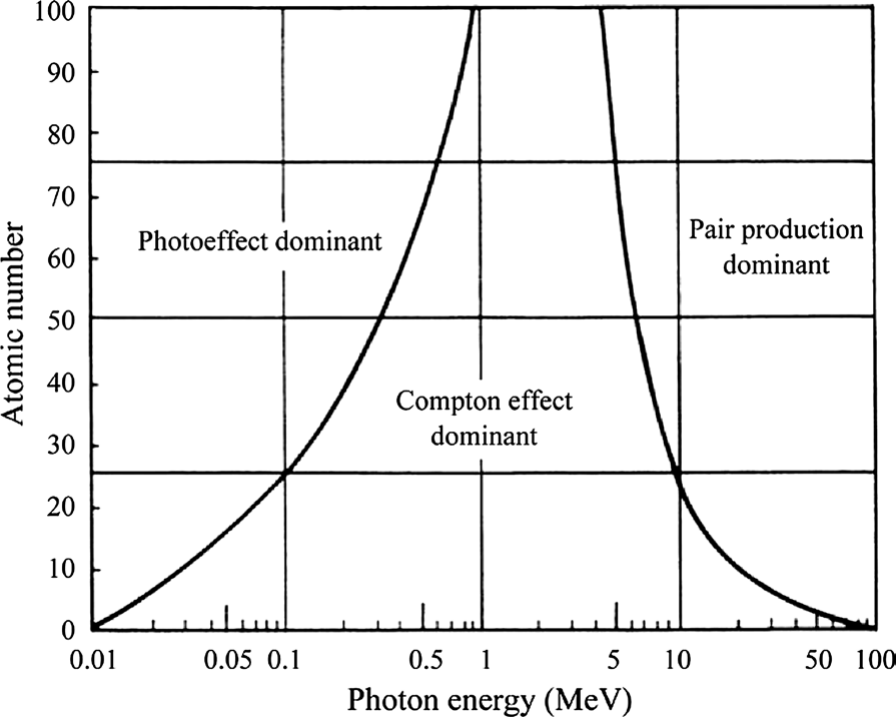
Predominating interaction versus photon energy for different atomic number absorbers (Reproduced with permission from reference: [143], copyright 2016 authors and Scientific Research Publishing Inc)

**Fig. 4:**
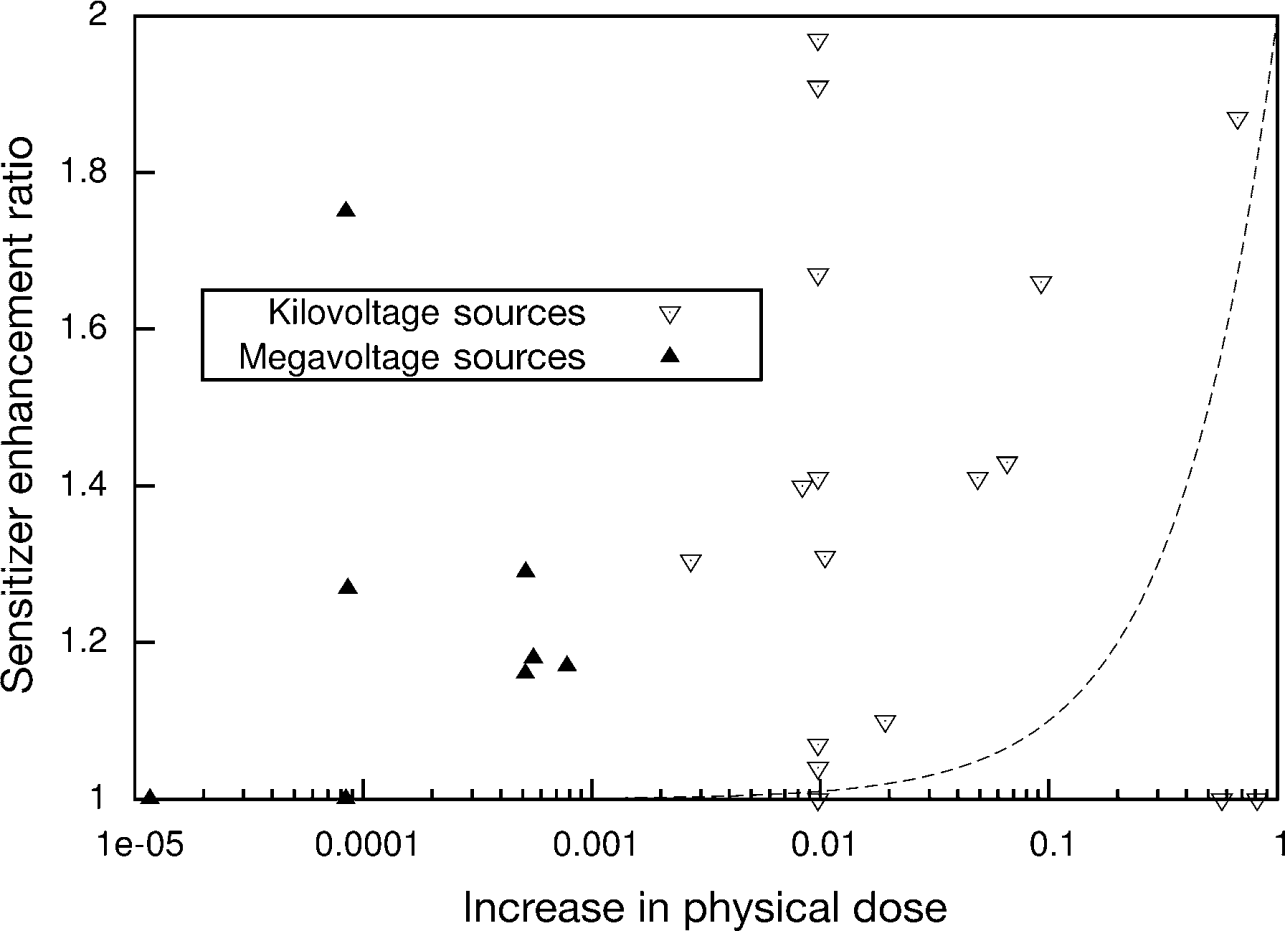
Comparison of predicted and observed experimental dose enhancement for gold nanoparticle studies. In horizontal axis, ‘Increase in physical dose’ refers to the ratio of the additional dose deposited by X-rays in the system due to the addition of GNPs to that which would be deposited in the absence of gold. Conducted energy source represented by kilovoltage (triangle) and megavoltage (black-up pointing triangle). The dashed line indicates the trend which would be followed if the sensitizer enhancement ratio directly involved with the predicted increases in physical dose. (Radiosensitization by gold NPs: effective at megavoltage energies and potential role of oxidative stress) Reproduced with permission from reference: [52], copyright 2013 Pioneer Bioscience Publishing Company

**Fig. 5:**
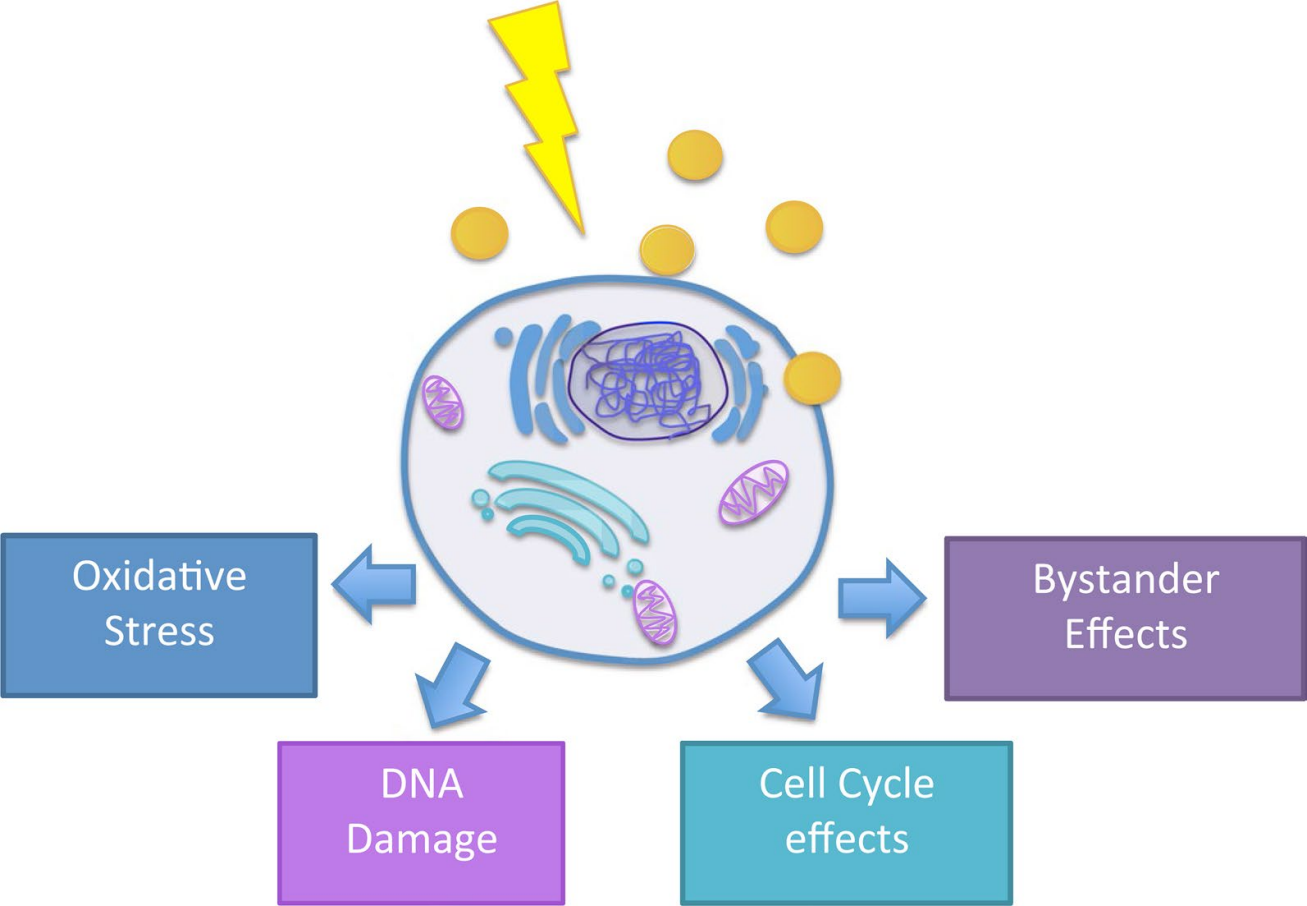
The biological mechanisms of GNP radiosensitization. There are several biological effect involved in GNP: oxidative stress, DNA damage, cell cycle, and bystander effects (Reproduced with permission from reference: [53], copyright 2017 The Author(s))

In clinical radiotherapy, 6–20 MeV energy is used because KeV energy has a relatively low tissue penetration depth [52]. Since Compton effect is dominant in the 6–20 MeV energy, the energy range is not suitable for radiosensitization by high-Z metal NPs, which is mostly based on the photoelectric effect (Fig. 3). However, the radiosensitization effect by high-Z metal NPs has been observed in both MeV and KeV energies (Fig. 4). This result was caused by the altered subsequent biological processes that occurred under radiation with high-Z metal NPs. The biological effect includes oxidative stress, DNA damage, cell cycle effect, and bystander effect (Fig. 5) [53].

#### ROS production

**Fig. 6:**
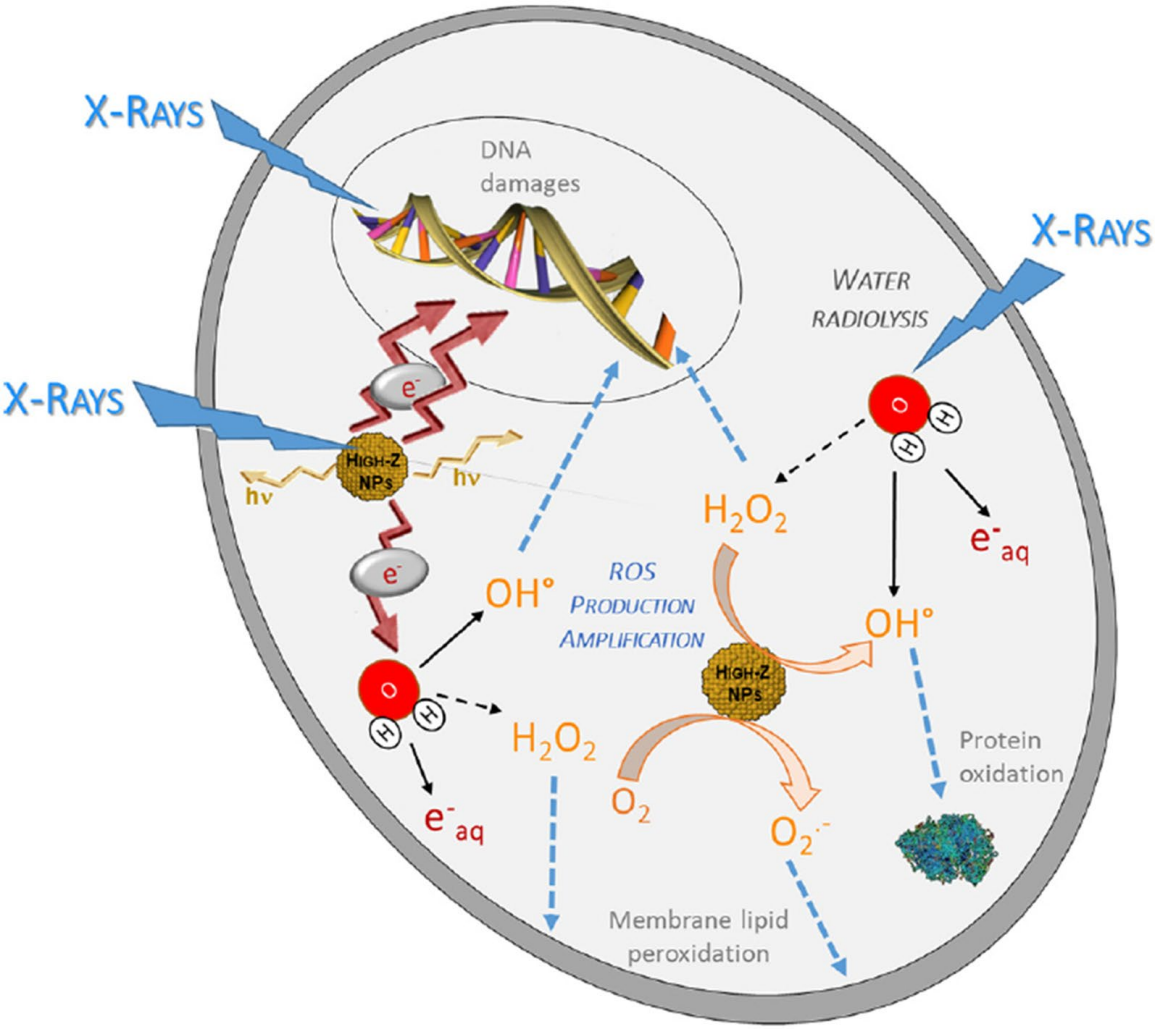
Biological mechanism of interaction between incident photons and high-Z NPs. Consequences of reactive oxygen species (ROS) production were generated from high-z nanoparticle through radiolysis water molecule. These ROS induce cell death via several effect (e.g. apoptosis, necrosis, mitotic cell death, autophagy, and permanent cell cycle arrest) and lead several types of defects such as base damages and protein modification (e.g. cross-linking, oxidation). Incident X-photons also damages DNA (e.g. single-strand breaks (SSBs), double-stranded breaks (DSBs)) by direct or indirect effect. With X-ray irradiation, amplified production of ROS and secondary electron from the high-Z nanoparticle result in cytotoxic enhancement on cells (Reproduced with permission from reference: [144], copyright 2018 Elsevier B.V)

Reactive oxygen species (ROS) mediated DNA damage is the primary mechanism of radiation-induced cell death. These include a group of oxidative species such as superoxide anion (O_2_^−^), hydroxyl radical (OH∙), hydrogen peroxide (H_2_O_2_), singlet oxygen (^1^O_2_), and hypochlorous acid (HOCl). The electrons generated by the interaction between radiation and material induce the formation of ROS, and ROS causes oxidative stress, DNA breakage, and apoptosis (Fig. 6) [54, 55]. High-Z metal NPs have been reported to induce significant levels of ROS and cause oxidative DNA damage [56, 57]. Pan et al. reported that 1–2 nm-sized gold NPs were highly toxic with an IC_50_ value of 30 to 56 μM. Multiple studies reported that ROS generation was enhanced when ionizing radiation was combined with high-Z metal NPs. Misawa et al. reported that a mixture of gold NP with sizes of 5–50 nm showed increased ROS by factors of 1.46 for OH∙ and 7.68 for O_2_^−^ under X-ray irradiation [58]. Recently, Choi et al. developed ROS sensor (dihydrorhodamine 123) attached gold NPs which enabled the direct evaluation of ROS generation by gold NPs. It was found that ROS generation by 6 Gy radiotherapy was enhanced in the ROS sensor attached gold NPs by a factor of seven compared to the ROS sensor alone [59]. Furthermore, Gadolinium (Gd) oxide NPs showed increased ROS production by a factor of 1.6 to 1.94 under 50 keV X-ray irradiation [60]. Taken together, these experimental results suggest that radiosensitization with high-Z metal NPs was mediated through enhanced ROS production.

#### Cell cycle effect

The biological effect of radiation differs depending on the different phases of the cell cycle. Specifically, S phase cells are the least radiosensitive, while mitotic cells are the most radiosensitive [61]. When the hydroxyl radical breaks the DNA double strands, ataxia-telangiectasia mutated (ATM) kinase is activated and phosphorylates p53 protein. P53 protein activated p21 protein which express a cyclin-dependent kinase (CDK) inhibitor, and then cell cycle arrest is occurred in G1 and G2 phases. Also, checkpoint kinase-1 (CHK-1) and checkpoint kinase-2 (CHK-2) phosphorylates cell division cycle 25 phosphatase, activating the CDK1-cyclinB and CDK2-cyclinE. It also causes cell cycle arrest in G1 or G2 phases [62–65].

It has been reported that high-Z metal NPs can induce radiosensitization through cell cycle arrest. Zhang et al. reported that octa-arginine modified gold NPs were able to induce cell cycle arrest in the G2/M phase and promoted cell apoptosis under 6 MV radiation [66]. Also, Roa et al. reported that glucose capped gold NPs could accelerate the G0/G1 progression resulting in accumulation of cells in the G2/M phase and enhanced radiation sensitivity in the radiation-resistant prostate cancer cell line [67]. High-Z metal NPs other than gold NPs has not been reported to cause cell cycle arrest effect under radiotherapy. However, a recent paper reported that Gd oxide nanocrystals induced cell cycle arrest and brought the higher radiotherapy effect under carbon ion therapy [68]. Above mentioned studies support that cell cycle arrest, which makes the cells more radiosensitive, can be promoted by high-Z metal NPs under radiotherapy conditions.

#### DNA damage and repair

Radiation-induced double-strand break (DSB) in DNA is the core mechanism of radiotherapy-mediated cell death. Several studies reported that high-Z metal NPs could induce DNA damage and reduce repair during radiotherapy. Chithrani et al. reported that 50 nm-sized gold NPs induced a higher number of DSB by γ-H2AX analysis during radiotherapy [69]. Another study showed that Gd-based NPs were able to reduce DNA repair and thus increased radiotherapy efficacy in glioblastoma cells. They observed the NPs-induced DNA damages even though the NPs were not localized in the nucleus [70]. Marill et al. reported that DNA damage was enhanced by radiotherapy-activated hafnium oxide NPs in the human colorectal cancer model compared to radiotherapy alone [71]. However, most of high-Z metal nanoparticles could not penetrate the cell as close as possible to affect the nucleus by physical dose enhancement. Thus, it is estimated that enhanced DNA damage is due to an increase in ROS production [72].

#### Enhanced bystander effects

The effect of radiotherapy can also be enhanced by intercellular communication. Cells that were not irradiated could be damaged by receiving signals from the adjacent irradiated cells. This process is called the bystander effect [73, 74]. As high Z-metal NPs have been shown to change cellular responses during radiotherapy, these altered responses could further affect cellular communication between irradiated and non-irradiated adjacent cells. However, there has not been direct evidence to prove the enhanced bystander effect by high Z-metal NPs during radiotherapy. High Z-metal NPs have been reported to alter protein synthesis and cytokine production; it is expected that high Z-metal NPs could enhance bystander effect during the radiotherapy. Fujiwara et al. reported that titanium dioxide NPs could induce higher levels of inflammatory cytokines production in lung and colon cancer models [75]. Furthermore, small airway epithelial cells exposed to gold NPs could induce protein expression in neighboring lung fibroblasts in co-culture systems. This study found that 47 proteins were upregulated, while 62 were downregulated in the fibroblasts receiving signals from the small airway epithelial cells incubated with gold NPs [76].

## High-Z metal NPs for radiotherapy of glioblastoma

### Gold nanoparticles

When gold NPs were irradiated with the wavelength larger than their sizes, d electrons were polarized by oscillation, resulting in surface plasmon resonance [77]. Using this property, research on cancer imaging and diagnostic biosensors is actively underway [78–82]. Also, gold NPs have unique optical properties that can be changed by their size and shape. For example, spherical gold NPs of about 25 nm size exhibit a unique ultraviolet absorption at 540 nm, and the absorption of the NPs tend to red-shift as the size increases [83]. Furthermore, if the absorption band of gold NPs is adjusted to be near-infrared, the NPs can be used for photothermal therapy. Trinidad et al. synthesized gold nanoshell, which can induce combined photothermal and photodynamic effects and eliminate cancer cells by generating ROS and oxidative stress [84]. In 2000, Herold et al. demonstrated the X-ray dose enhancement effects by gold NPs using a gold microsphere solution in Chinese hamster ovary cells (CHO-K1), mouse breast cancer cells (EMT-6), and human prostate cancer cells (DU-145) [85]. After that, studies to evaluate the radiosensitizing effect of gold NPs, as well as of other types of high Z metal NPs, were conducted in multiple types of cancer, including GBM. A summary of the enhanced radiosensitization in GBM using high-Z metal NPs is provided in Table 1.

**Table 1:** Radiosensitizing effect of high-Z metal NPs in glioblastoma model

High-Z metal	Particle name (commercial or by the authors)	Coating	Hydrodynamic Size	Functionalization	Injection mode	Concentration of NPs administerated in vivo	Concentration of NPs in tumor	Radiation source	In vitro/In vivo	Radiation Energy	Radiation dose	Cells and tumor models	Radiosensitizing effect	Refs
Gold	GNPs	PEG	23 nm	None	i.v.	1.25 g Au/kg	3.7% ID/g Tissue of gold	Small Animal Radiation Research Platform	In vitro	150 kVp	0 ~ 6 Gy	U251	SER = 1.3	Johet al. [88]
									In vivo	175 kVp	20 Gy	U251 orthotopic mouse model	Median survival time: RT vs. NP + RT: 14 vs. 28 days	
	AuNPs-Pt	PEI	50 nm	Cisplatin	In vitro only	In vitro only	In vitro only	Cesium-137 beam radiator	In vitro	1 Gy per minute	10 Gy	S1, S2, SP56 (Patient-derived GBM cell line)	NP + RT: Complete cancer ablation RT: Recurrence	Setua et al. [90]
	BSA-AuNPs	BSA	28 nm	None	i.v.	1.3 mg/mL BSA-AuNPs	2%ID/g Tissue of gold	N.R.	In vitro	160 kVp X-ray	0 ~ 8 Gy	U87	SER = 1.37	Chen et al. [89]
									In vivo	160 kVp	5 Gy	U87 subcutaneous mouse model	Tumor weight: RT vs. NP + RT = 0.15 g vs. 0.1 g	
	pAuNTs (Au nanotriangles)	None	61.51 nm (Narrow edge length)	PEG	i.v.	2.7 mg/kg pAuNTs	5000 ng/g (Au/tissue)	XRAD 320 orthovoltage irradiator	In vitro	250 kVp X-rays	0 ~ 6 Gy	MCF-7, U87MG	Maximum reduction of surviving fraction: 3-4 Gy	Bhattarai et al. [9]
									In vivo		N.R	U87MG subcutaneous mouse model	DEF = 2.67 Time to tripling of tumor: RT vs. NP + RT = 3 days vs. 8 days	
Gadolinium	GdBN	Polysiloxane shell	3 nm	None	In vitro only	In vitro only	In vitro only	Gamma radiation, Cobalt source (^60^Co) at CEA	In vitro	1.25 MeV (1 Gy per minute)	0–7 Gy	U87	Enhancing factor_2 Gy_ = ~ 23%	Stefancikov et al. [105]
	GdBNs	Polysiloxane shell	3 nm	None	In vitro only	In vitro only	In vitro only	Gamma ray, ^60^Co irradiator	In vitro	1 Gy per minute	0–4 Gy	U87	Survival fraction: RT vs. NP + RT = 0.81 vs. 0.55 (1 Gy) RT vs. NP + RT = 0.42 vs. 0.37 (4 Gy)	Stefancikova et al. [70]
	Gd-based particle	Polysiloxane shell	3 nm	None	In vitro only	In vitro only	In vitro only	^137^Cs source irradiator, Pantak Therapax STX 150 apparatus	In vitro	660 keV	0–8 Gy	U87	DNA DSBs of GdNP + 5 Gy: 84% versus RT only Cell killed by NP sensitizing effect (2 vs. 5 vs. 8 Gy: 12.5% vs. 25% vs. 37%)	Le Duc et al. [103]
Silver	AgNPs	Citrate	26.75 nm	None	i.t.	10 μg (mass-AgNPs) 5.48 μg (mol-AgNPs)	N.R	linear accelerator	In vitro	6 MV X-rays (200 cGy per minute)	0–8 Gy,	U251	SER: mas-AgNPs: 1.64 mol-AgNPs: 1.44	Liu et al. [115]
								vertical accelerator	In vivo	6 MV X-rays	8 Gy	U251 orthotopic mouse model	MST: RT vs. mas-AgNP + RT vs. mol-AgNP + RT = 35 days vs. 62 days vs. 51 days)	
	AgNPs	PVP	88.6 nm	None	i.t.	10 μg or 20 μg	N.R	Vertical beam with linear accelerator	In vivo	6 MV X-rays (200 MU per minute)	10 Gy	C6 orthotopic rat model	MST: IR vs. 10 μg AgNPs + RT vs. 20 μg AgNPs + RT = 24.5 vs. 33.5 vs. 37.0 days	Liu et al. [114]
Iron oxide Iron oxide	cetuximab-IONPs	Amphiphilic polymer	Core size: 10 nm	Cetuximab	CED	0.3 mg/mL	N.R	X-RAD	In vitro	320 kV, 10 mA (1.2 Gy per minute)	10 Gy	U87MGEGFRvIII	Cetuximab-IONP + RT: higher density of DNA DSBs, enhanced intracellular ROS production	Bouras et al. [37]
									In vivo	320 kV, 10 mA (1.2 Gy per minute)	10 Gy x 2 (24, 72 h)	U87MGEGFRvIII orthotopic mouse model	MST: RT vs. cetuximab-IONPs + RT = 15 vs. 60	
	SPION-cmHsp70.1	Dextran	54.8 nm	Monoclonal cmHsp70.1 antibody	i.v.	2.5 mg/kg	N.R	N.R	In vitro	N.R	0–20 Gy	C6, K562, HeLa	Dramatical increase in surface density of Hsp 70 by radiotherapy	Shevtsov et al. [122]
								Monochromatic X-ray beams, SARRP irradiator,	In vivo	225 kVp	10 Gy	C6 orthotopic rat model	SPION-cmHsp70.1 uptake was enhanced by radiotherapy	
Bismuth oxide	Bi_2_O_3_ NP	None	50–70 nm	None	In vitro only	In vitro only	In vitro only	Nucletron Oldelft Therapax, Elekta Axesse™ LINAC	In vitro	125 kVp, 10 MV	0–8 Gy,	9L	SER (125kVp) = 1.48 SER (10 MV) = 1.25	Stewart et al. [123]
TIONTs	TiONts	None	10 nm	None	In vitro only	In vitro only	In vitro only	linear photon accelerator (clinic600, Varian)	In vitro	N.R	0–10 Gy	SNB-19, U87MG	SF_2 Gy_ (SNB19): RT vs. NP + RT = 0.36 vs. 0.18 SF_2 Gy_ (U87MG): RT vs. NP + RT = 0.60 vs. 0.43	Mirjolet [124]
Tantalum pentoxide	Tantalum pentoxide NSPs	None	56 nm	None	In vitro only	In vitro only	In vitro only	Clinical LINAC	In vitro	10 MV X-rays	0–8 Gy	9L	SER = 1.33	Brown et al. [113]
Gold, Iron oxide	GSMs	PEG-PCL copolymer	~ 100 nm	None	i.v.	300 mg Au/kg	Brain tumor: 1.7% ID/g, flank tumor: 2.2% ID/g	Small Animal Radiation Research Platform (SARRP)	In vitro	150 kVp, 0.5 mA	4 Gy	U251, U373	DNA DSBs change (U251) = 1.8-fold DNA DSBs change (U373) = 2.4-fold	Sun et al. [127]
								N.R	In vivo	N.R	N.R	U251 orthotopic mouse model	GSM uptake (Normal brain:brain tumor = 1:97)	
BaYbF_5_:2% Er^3+^	UCA-RGD	None	22 nm	RGD peptide	i.v.	70 mg Yb/kg (7 mg Yb/mL)	7.5% ID Yb/g	Siemens Primus clinical linear accelerator	In vitro	6 MeV	8 Gy	U87MG	Cell uptake is enhanced by RGD peptide	Xing et al. [126]
									In vivo	6 MeV	8 Gy	U87MG Subcutaneous mouse model	Tumor volume shrinkage of UCA-RGD + RT group = 35%	

PEG: polyethylene glycol; PEI: polyethylenimine; PVP: polyvinylpyrrolidone; cmHsp70.1: Hsp70.1-specific antibody; PEG-PCL: polyethylene glycol-polycaprolactone; i.v: intravenous injection; i.t: intratumoral injection; CED: convection-enhanced delivery; RT: radiation therapy; IR: Ionizing radiation; SER: sensitizer enhancement ratio; DEF: dose enhancement factor; DSBs: double-stranded breaks; MST: Median survival time; SF: Survival fraction; ROS: reactive oxygen species

**Fig. 7:**
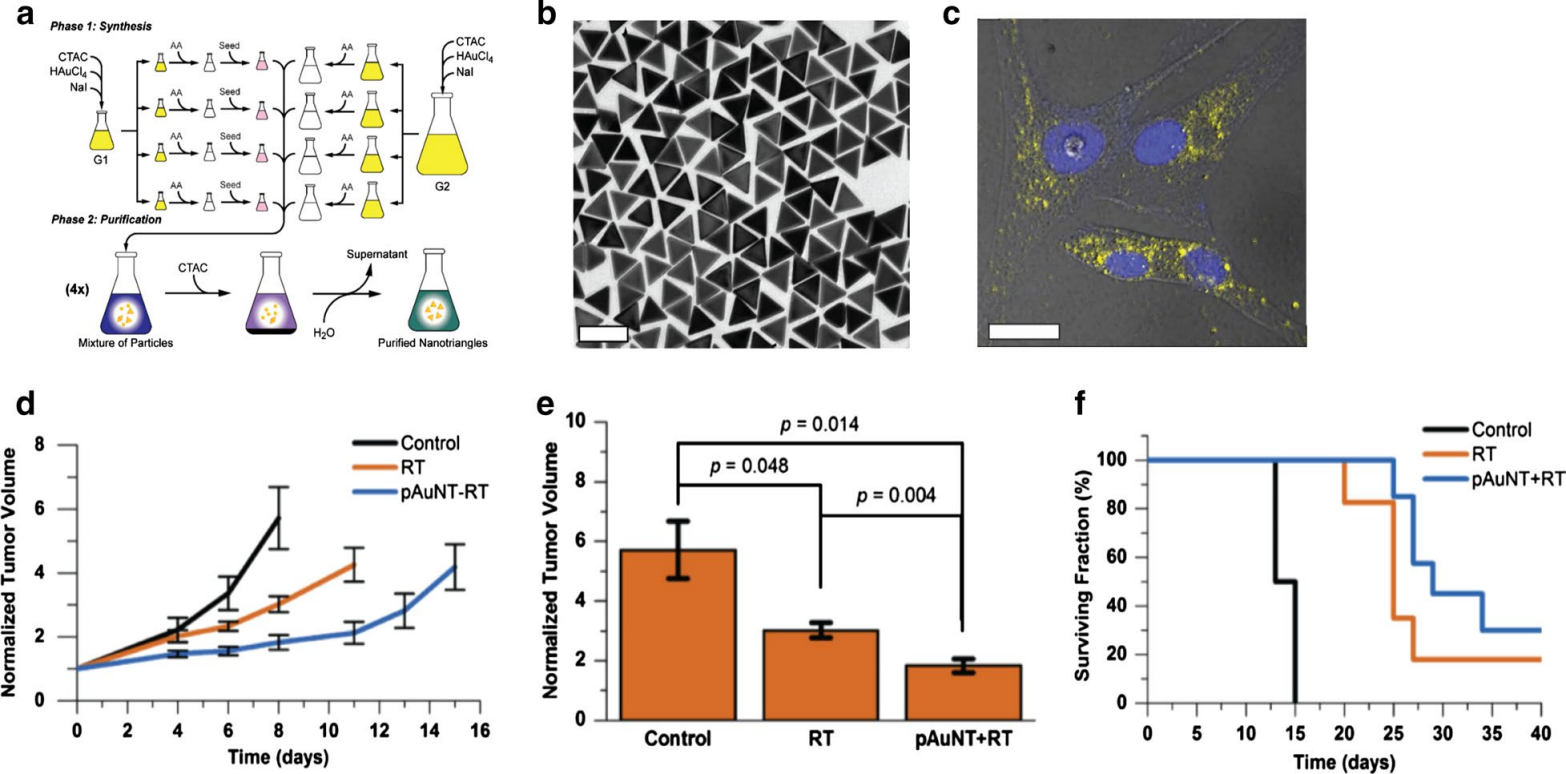
Radiation sensitizing effect of gold nanotriangle. **a** Schematic of synthesis gold nanotriangles with large-scale. **b** TEM image of well-purified CTAC-capped AuNTs. Scale bar is 100 nm. **c** PEGylated AuNTs uptake by adherent cells (U87MG cells). Blue: DAPI, Yellow: AuNTs. **d** AuNTs delayed the tumor growth following subcutaneous U87MG xenografts. The comparisons include 3 groups: with vehicle only (control); with radiotherapy alone (RT); and PEGylated AuNTs with radiation (pAuNT + RT). **e** The difference between above treatment in tumor volume. **f** RT + pAuNTs treated mice displayed an improving tendency of actuarial survival (p = 0.05) (Reproduced with permission from reference: [9], copyright 2018 PMC)

Joh et al. synthesized 23-nm size gold NPs with increased biocompatibility using the Turkevich method and PEGylation. The gold NPs significantly improved radiation sensitivity of the GBM model in vitro and in vivo. The human GBM cells treated with radiotherapy and gold NPs showed 1.7-fold higher level of DNA damage than the cells treated with radiotherapy alone. Furthermore, the orthotopic GBM mouse model treated with gold NPs and radiotherapy demonstrated a twofold prolonged survival time than those treated with radiotherapy alone. Delivery of pharmaceutics, including NPs, to the brain is challenging because of the blood–brain barrier (BBB) [86, 87]. Joh et al. found that the accumulation of gold NPs was higher in the brain hemisphere with GBM than in the other hemisphere without GBM, probably due to disrupted BBB in GBM. In addition, the gold NPs uptake in GBM could be further enhanced when the NPs were injected after radiotherapy, indicating that BBB can be further destroyed by radiotherapy [88]. There have been multiple methods to improve BBB penetration of intravenously injected NPs, such as focused ultrasound-mediated BBB disruption, cell-penetrating peptide-mediated, receptor-mediated, and shuttle peptide-mediated methods [86]. For the radiosensitization in GBM, these methods should be considered to maximize the effect of NPs. Chen et al. performed the clonogenic assay after radiotherapy using 28-nm size bovine serum albumin (BSA)-capped gold NPs in U87 cells. The number of colonies after radiotherapy was smaller when the cells were incubated with the gold NPs during radiotherapy than radiotherapy alone, and the calculated sensitizer enhancement ratio (SER) was 1.37. Moreover, the DNA damage and cell apoptosis were stronger in the cells treated with gold NPs and radiation than in the cells treated with radiation alone. In vivo radiosensitizing effect was assessed in subcutaneous mice model. Radiotherapy could inhibit tumor growth, and the combination of radiotherapy and BSA capped gold NPs led to significantly better tumor regression, with statistically significant difference [89]. In 2017, Bhattarai et al. synthesized a large-scale mPEG-SH functionalized gold nanotriangles (gold NTs) (Fig. 7A, B). Since the higher cellular uptake of NPs can enhance the radiosensitization effect, they compared the cellular uptake efficiency of the gold NTs with 2 kDa, 5 kDa, 10 kDa, 20 kDa, and 30 kDa mPEG-SH. The gold NTs with 5 kDa mPEG-SH showed the highest cellular uptake among the tested NTs. Further, they found that the endocytosed gold NTs were distributed near the nucleus of U87MG cells (**Fig.** **7C**). Moreover, mice treated with the radiotherapy and gold NTs showed significantly better tumor regression and prolonged survival than mice treated with radiotherapy alone with a dose enhancement factor of 2.67 (Fig. 7 D– F) [9]. Setua et al. developed cisplatin-tethered gold NPs (gold NP-Pt) for concomitant chemo-radiotherapy of GBM. Gold NP-Pt with radiotherapy showed a synergistic treatment effect in patient-derived GBM cells. The authors confirmed that the GBM cells treated with gold NP-Pt and radiotherapy showed a significantly higher degree of DNA damage and lower cell survival in vitro than GBM cells treated with the combined radiotherapy and gold NPs and those treated with radiotherapy alone [90].

### Gd-based nanoparticles

Gadolinium is a lanthanide element which has eight unpaired electrons with the preferable oxidation state of +3 [18]. This unique electronic configuration of Gd changes the signal intensity of the longitudinal relaxation rate of water protons (1/T_1_) to the higher than the one in the transverse rate (1/T_2_) in magnetic resonance imaging (MRI). The relaxivity, which is the feature to change the relaxation rate of the surrounding water molecule, is defined as the difference in relaxation rate normalized by the concentration of contrast agent. Gadolinium chelates can be used as a T1 contrast agent that enhances tissue contrast in T1 weighted MRI [91]. Diverse structures of Gd complexes like polymer, dendrimer, and liposome are studied to achieve higher relaxivity with different rotational diffusion, water exchange, distance, and relaxation rate [91, 92].

Motexafin Gd (MGd) is the most studied Gd theranostic anti-cancer agent. MGd is a redox mediator with an aromatic macro cycle structure that generates ROS and inhibits tumor growth promoters [93, 94]. In addition, Gd cation of MGd augments signals in T1-weighted MRI imaging. MGd is studied for its ability to target brain tumors such as GBM, and brain metastases originating from lung cancer [95]. MGd showed a good targeting efficiency to nuclei of glioblastoma cancer cell lines. Gadolinium could be infiltrated to the cancer cell nuclei by MGd vehicle [96]. Furthermore, MGd showed an enhanced cytotoxic effect when combined with radiation in various clinical studies [97–99]. Based on these studies, research on radiosensitization and theranostics using Gd-based NPs is growing [100].

**Fig. 8:**
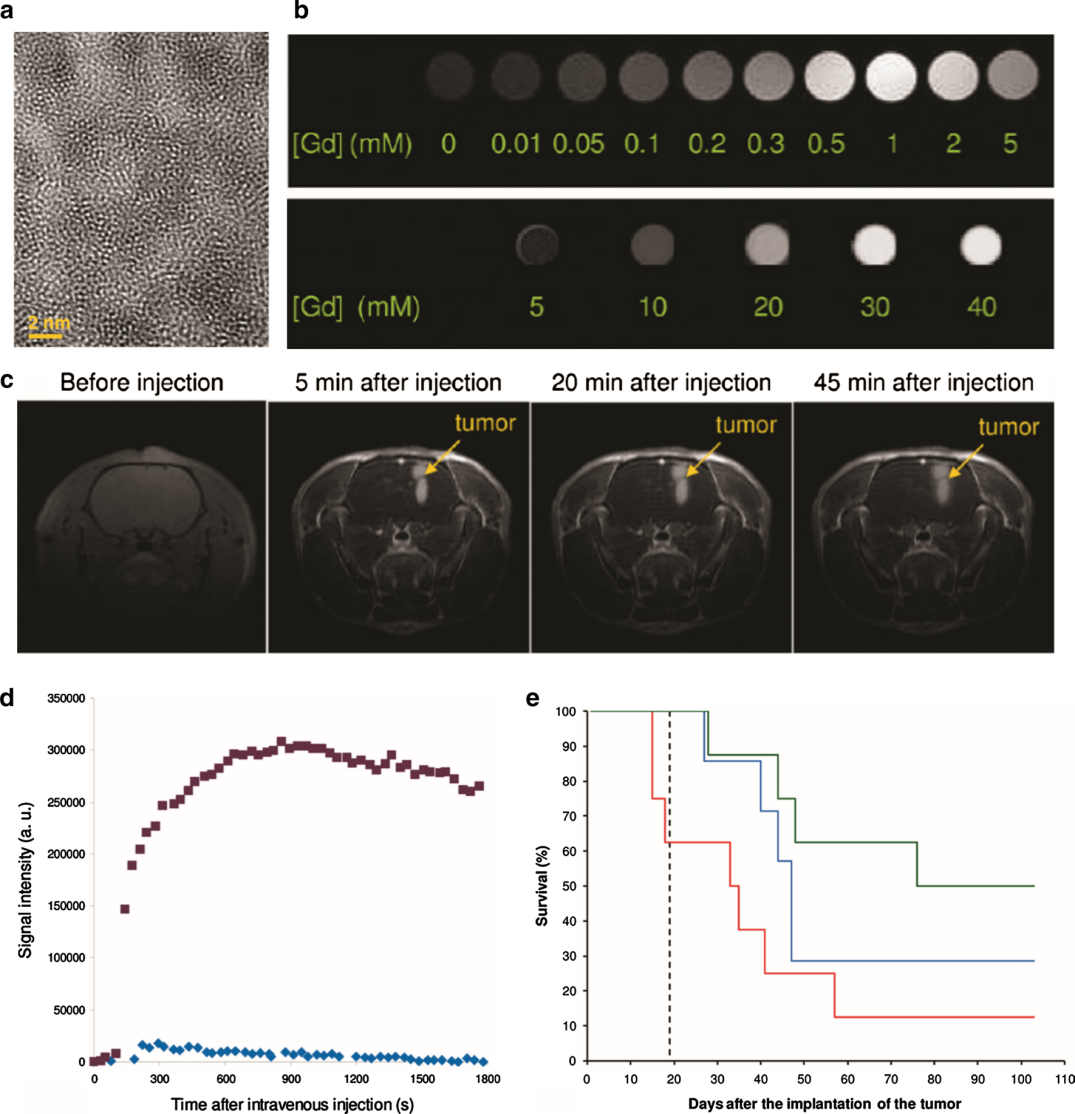
The survival improvement of brain tumor bearing rats with combination of GBN and MRT. **a** HR-TEM image of encapsulated gadolinium oxide NPs in polysiloxane shell. Scale bar is 2 nm. **b** The enhancement of gadolinium with proportion to concentration as the contrast. Upper: T_1_-weighted, bottom: SPCT. **c** Brain images of 9LGS-bearing rat by T_1_-weighted at various time points. **d** MRI signal in tumor (purple) and in normal tissue of equivalent surface (blue) in process of time. **e** Survival cureve of 9LGS-bearing rate. Black dash curve represents without treatment group (n = 4). Blue curve, red curve and green curve represent only treated by MRT (n = 7), treated by MRT 5 min (n = 8) and 20 min (n = 8), respectively. The survival curve was filled out up to 103 days after tumor implantation. MRT: microbeam radiation therapy (Reproduced with permission from reference: [103], copyright 2011 American Chemical Society)

Radiosensitization using Gd-based NPs in GBM is attractive because Gd-based NPs can be used as an MRI contrast agent, and MRI is the image of choice for the management of GBM [101]. Activation and Guidance of Irradiation by X-ray (AGuIX) are theranostic NPs that can be used as a radiosensitizers and contrast agents for MRI. The NPs are composed of polysiloxane network core covered by Gd chelates. AGuIX NPs have very small hydrodynamic diameters (< 5 nm) and biodegradability [102]. Le Duc et al. synthesized AGuIX NPs composed of Gd oxide core and polysiloxane shell functionalized by DTPA chelator for radiosensitization and MR imaging (Fig. 8a, b). In orthotopic 9LGS gliosarcoma rat models, the NPs could be targeted to the tumor passively and cleared efficiently through the kidneys. Gadolinium concentration, measured by ICP-MS, was two-fold higher in the hemisphere with GBM than in the hemisphere without GBM at 20 min after the injection. In addition, the tumor-bearing rat treated with radiotherapy and the NPs showed significantly longer median survival time than the rats treated with radiotherapy alone (Fig. 8e). The Gd component could be excreted through urine up to 30% during the first hour after intravenous injection of the NPs. Moreover, the tumor could be well visualized in T1-weighted MRI after intravenous injection of the NPs, which confirms the ability of the NPs as a T1 contrast agent (Fig. 8c, d) [103]. The same group modified the NPs by changing the chelator from DTPA to DOTAGA to prevent any release of Gd from the NPs. The NPs have about 2.1 nm hydrodynamic size and are biodegradable in diluted media and BSA. The circulating half-life of the NPs was about 20 min, and the NPs were excreted efficiently through the kidneys. The authors compared the ability as an MRI contrast and radiosensitizing agent between the NPs and DOTAREM^®^, a commercially available Gd based contrast agent. The AguIX showed a better MRI contrast in healthy animals because the NPs had a longer circulation time compare to DOTAREM^®^. In orthotopic 9LGS gliosarcoma rat models, the median survival time after microbeam radiation therapy (MRT) with AGuIX NPs was 102.5 days, which was longer than MRT alone (44 days) and MRT with DOTAREM^®^ as a radiosensitizer (43 days) [104]. The radiosensitizing mechanism of AGuIX NPs was also investigated in U87 glioblastoma cells. The NPs were located in the cytoplasm (especially in lysosomes) but did not enter into the cell nucleus. The cell-killing effect by radiotherapy was enhanced by 23% at 2 Gy irradiation [105]. Another study also reported that the radiosensitization of AGuIX NPs mainly resulted from cytoplasmic events rather than from nuclear DNA damages [70]. AGuIX NPs are now under clinical trials for the treatment of multiple brain metastases (NCT04094077), and advanced cervical cancer (NCT03308604).

There have been studies reporting modified AGuIX NPs for multimodal imaging or improved therapeutic effect. ^68^Ga-radiolabeled AGuIX with the novel chelator 2,2′-(7-(1-carboxy-4-((2,5-dioxopyrrolidin-1-yl)oxy)-4-oxobutyl)-1,4,7-triazanonane-1,4-diyl)diacetic acid) (NODAGA) NPs (^68^Ga-labeled AGuIX@NODAGA NPs) were developed for dual PET/MR imaging. The hydrodynamic size of the NPs was 4.3 nm; thus, the NPs were suitable for renal elimination. The NPs showed a moderate passive tumor targeting ability in U87MG tumor-bearing mice (1.03% ID/g, 30 min after injection). They confirmed that ^68^Ga-labeled AGuIX@NODAGA NPs were not excreted through the liver but through kidneys by the metabolite and biodistribution studies. The tumor uptake was well visualized in both MR and PET imaging after intravenous injection of the NPs [106]. Another study showed IR-783 functionalized AGuIX NPs for PET/MRI/optical imaging. The NODAGA chelator was used for radiolabeling and IR-783 for optical imaging [107]. For vascular-targeted photodynamic therapy (VDT), newly synthesized AGuIX type nanoplatform using photosensitizer and KDKPPR peptide moiety targeting neuropilin-1 (NRP-1), which is highly expressed in the tumor vasculature, were developed (AGuIX@PS@KDKPPR). In U87 orthotopic mice tumor models, the tumor contrast was enhanced in the MRI after injection of the NPs. In human umbilical vein endothelial cells (HUVEC), AGuIX@PS@KDKPPR showed a higher VDT effect compares to that of AGulX@PS@Scramble, indicating the enhanced therapeutic effect by the targeting moiety. In U87 subcutaneous mice models, in vivo vessel specific uptake of the NPs was observed [108].

### Other nanoparticles

**Fig. 9:**
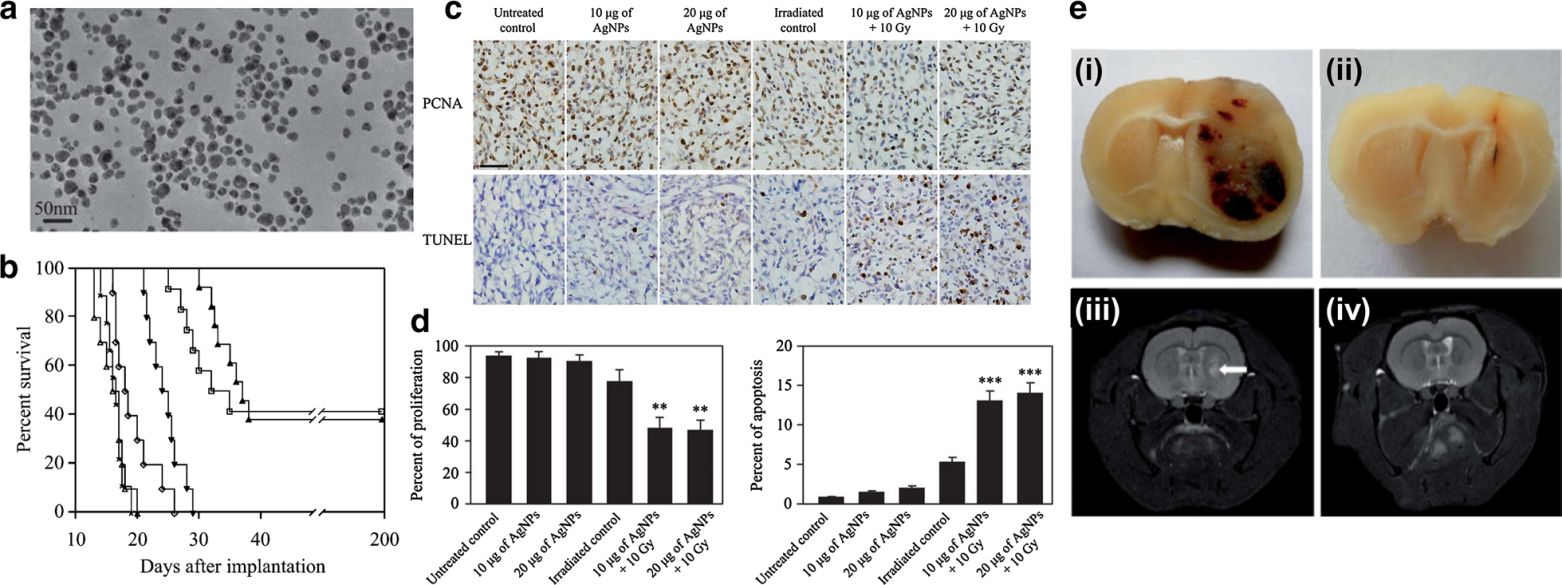
Radiation enhancement effects of PVP-coated AgNP. **a** TEM characterization of silver NPs. Scale bar is 50 nm. **b** Kaplan–Meier survival curve for C6 glioma-bearing rats with intratumoral administration of AgNPs. Six groups were involved as comparison: untreated animals (x); 10 μg of AgNPs alone (white triangle); 20 μg of AgNPs alone (white diamond); irradiated control (black down-pointing triangle); 10 μg of AgNPs + 10 Gy (white triangle); and 20 μg of AgNPs + 10 Gy (black up-pointing triangle). **c** Proliferating cell nuclear antigen (PCNA) or terminal deoxynucleotidyl transferase-mediated deoxyuridine triphosphate nick and labeling (TUNEL) staining of each comparison group. **d** Proliferation and apoptosis were quantitatively analysed into vertical bar charts, individually. **e** The brain images of glioma-bearing. (i) and (ii) represent frontal slices of untreated control in extremis and rat surviving for 200 days. (iii) and (iv) represent T_2_-weighted MR images of well-implanted tumor after 7 days and rat surviving for 200 days which exhibits absence of tumor, respectively (Reproduced with permission from reference: [114], copyright 2013 Royal Society of Chemistry)

Aside from the high-Z metal NPs mentioned above, silver NPs have also been widely investigated in biomedical applications. Clinically used products that contain silver NPs are mainly a sort of wound dressing and catheters [109]. Further, many studies investigated the anticancer effect and cytotoxicity of silver NPs [110, 111]. Silver NPs induce cytotoxicity via mechanisms such as apoptosis, ROS generation, inhibition of mitochondria function, membrane leakage, and membrane lipid peroxidation [112]. Liu et al. firstly reported the radiosensitizing effect of silver NPs in glioma cells. They synthesized silver NPs of diameter 20, 50, and 100 nm and compared the radiosensitizing effects of the NPs. The authors found that the smaller size of NPs had a stronger radiosensitizing effect in the U251 glioma cell line. Similar results were drawn in C6 and SHG-44 cell lines [113]. The same group investigated the radiosensitizing effect in vivo using 20 nm-sized silver NPs (Fig. 9a). Silver NPs were intratumorally injected in orthotopic C6 glioma-bearing rats treated with MV energy radiotherapy. The median survival time was significantly longer in mice treated with silver NPs and radiotherapy compared to that in mice treated with radiotherapy alone (18 vs. 37 days) (Fig. 9b, e). The authors found that the mechanism of radiosensitization was combined with anti-proliferative and apoptotic effects (Fig. 9c, d) [114]. Liu et al. also reported that the radiosensitizing effect of silver NPs was superior to that of gold NPs in vitro and in vivo. They compared the radiosensitizing effect between 15 nm-sized citrated coated gold NPs and silver NPs. The silver NPs showed significantly better growth inhibition rate in U251 glioma cells than the gold NPs (dose enhancement ratio: 1.64 vs. 1.23). In the orthotopic U251 glioma mice model, the median survival time was significantly longer in mice treated with silver NPs and radiotherapy than in mice treated with gold NPs and radiotherapy (61.7 days vs. 43.1 days). The silver NPs induced a higher level of proapoptotic activity and autophagy compared to gold NPs, which explains the superior radiosensitizing effect of silver NPs [115].

**Fig. 10:**
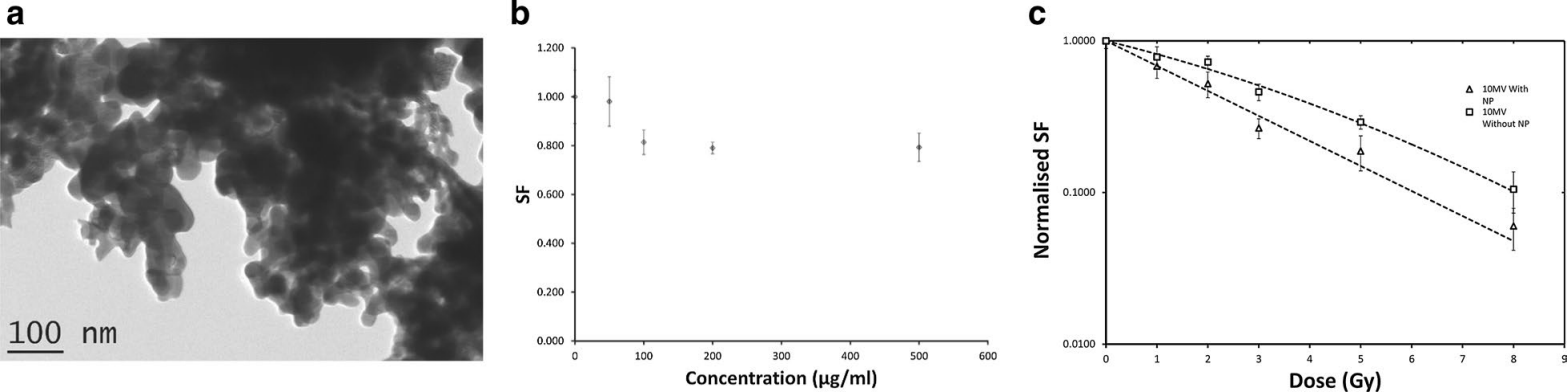
Dose enhancement effect of Ta_2_O_2_ on radioresistant cancer cells. **a** Tantalum pentoxide NSPs imaging by HR-TEM. Scale bar is 100 nm. **b** survival fraction of tantalum pentoxide NSPs with various concentration. 9L cells were exposed 0-500 μg/mL over 24 h. **c** Cell survival curve of 10 MV X-ray irradiation with dose variation (n = 3) (Reproduced with permission from reference: [117], copyright 2013 WILLEY-VCH Verlag GmbH & KGaA, Weinheim)

Tantalum metal is a high-Z metal with an atomic number of 73. Tantalum pentoxide (Ta_2_O_5_) NPs were reported to be biocompatible and capable of drug loading [116]. In 2013, Brown et al. first reported the utility of Ta_2_O_5_ nanoceramics as a radiosensitizer (Fig. 10a). The 50–70 nm-sized tantalum oxide NPs showed the sensitizer enhancement ratio (SER) of 1.33 at a 10 MV x-ray photon beam in 9L gliosarcoma cell line (Fig. 10b, c) [117]. The same group also reported the dose enhancement effect of the NPs under a synchrotron beam with the energy range of 50–150 keV in 9L gliosarcoma cells [118].

**Fig. 11:**
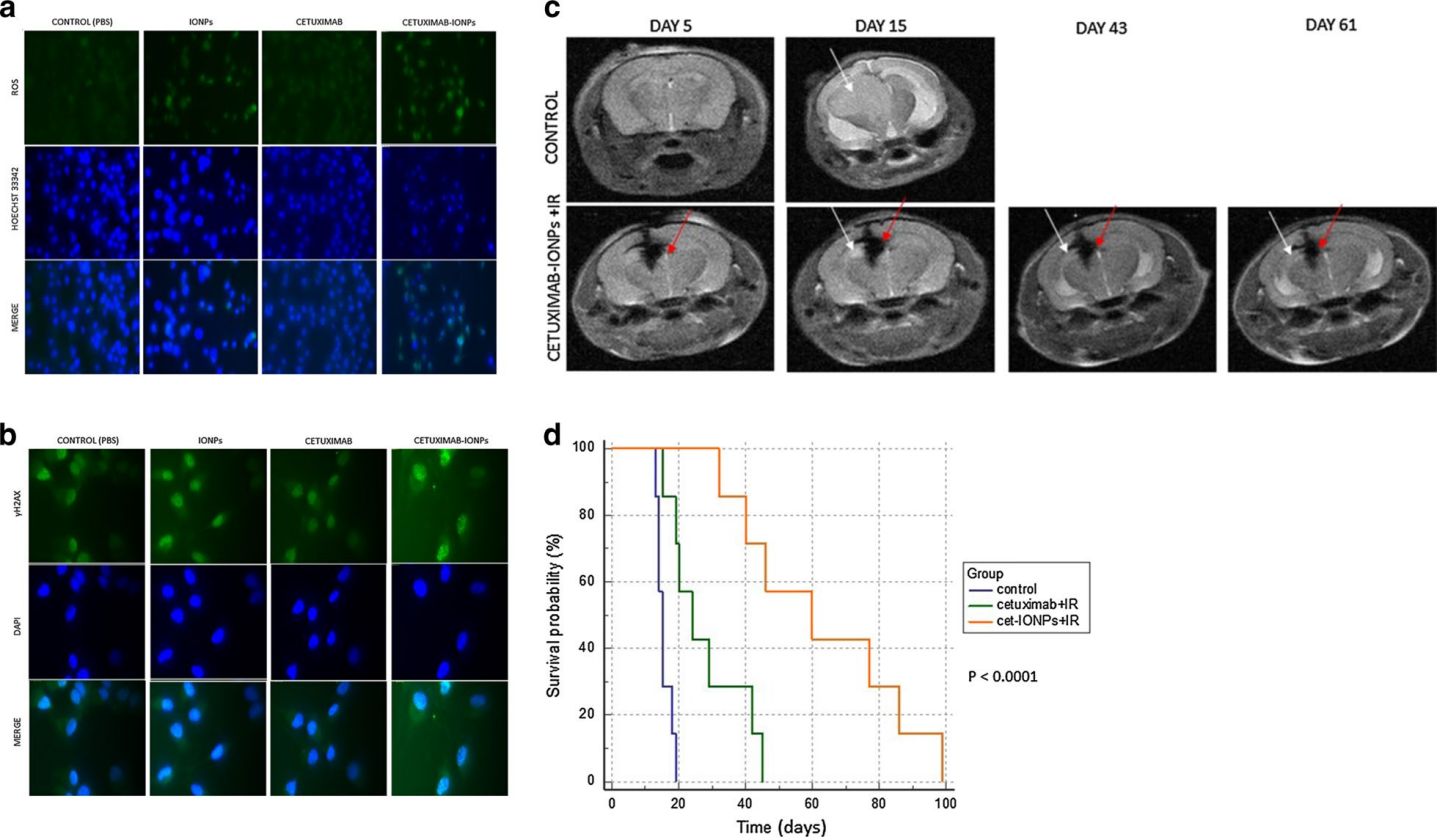
Radiosensitivity enhancement of radiosesistant human GBM by cetuximab-IONP treatment. **a** Representative immunofluorescence image of U87MGEGFRvIII cells after treatment of four comparison groups (0.3 mg/mL of PBS, IONPs, cetuximab and cetuximab-IONPs) with ionizing irradiation (IR) dose of 2 Gy and incubation for 24 h. **b** ROS detection of above treatment with ionizing irradiation dose of 10 Gy and incuation for 24 h. **c** Hypointense T_2_-weighted MRI images of control and cetuximab-IONPs + IR groups. White arrows: EGFRvIII-expressing human xenograft, red arrows: convection-enhanced delivery(CED) of cetuximab-IONPs. **d** Kaplan–Meier survival curve for U87MG EGFRvIII inplanted mice. Three groups are involved as comparison: no treatment (control); combination of cetuximab and IR treatment (cetuximab + IR); and cetuximab-IONPs with subsequent IR (cet-IONPs + IR). Concentration of cetuximab was 0.3 mg/mL and IR dose were 10 Gy × 2 (Reproduced with permission from reference: [37], copyright 2015 Springer Science + Business Media New York)

Superparamagnetic iron oxide NPs (SPIONs) have been investigated for hyperthermia treatment and MR imaging of GBM [119, 120]. The efficacy and biocompatibility of combined magnetic hyperthermia treatment using intratumorally injected SPION and radiotherapy was tested in patients with recurrent GBM [121]. Antibody conjugated iron oxide NPs have also been developed to improve tumor targeting [37, 122]. Shevtsov et al. conjugated heat shock protein Hsp70 specific antibody to dextran-coated SPIONs. Hsp70 was expressed in malignant cells, and the expression level was enhanced when tumor cells were irradiated by ionizing radiation. Synchrotron radiation with 54.8 nm-sized SPION-cmHsp70.1 NPs improved NPs uptake in the C6 glioma cell line. Furthermore, in orthotopic C6 glioma models, MRI and ex vivo biodistribution analysis revealed that SPION-cmHsp70.1 NPs tumor uptake was enhanced after radiotherapy. The enhanced uptake was due to the increased Hsp70 expression in tumor cells after the radiotherapy [122]. Bouras et al. synthesized cetuximab, epidermal growth factor receptor (EGFR) specific antibody, conjugated to iron oxide NPs. The authors confirmed that the radiosensitizing effect was higher in cetuximab—iron oxide NPs compared to that in non-conjugated iron oxide NPs by testing the extent of apoptosis, DNA DSBs formation, and ROS generation in a U87MG cell line overexpressing the EGFRvIII deletion mutant (U87MGEGFRvIII) (Fig. 11a, b). Further, the authors delivered cetuximab-conjugated iron oxide NPs by convection-enhanced delivery in orthotopic EGFRvIII expressing GBM mice model. The mice treated with NPs and radiotherapy elicited extended survival compared to the mice treated with cetuximab and radiotherapy (Fig. 11c, d) [37]. The radiosensitizing effect was also reported in another type of metal NPs including bismuth oxide NPs [123], titanate nanotubes [124], and hydroxyapatite NPs [125].

**Fig. 12:**
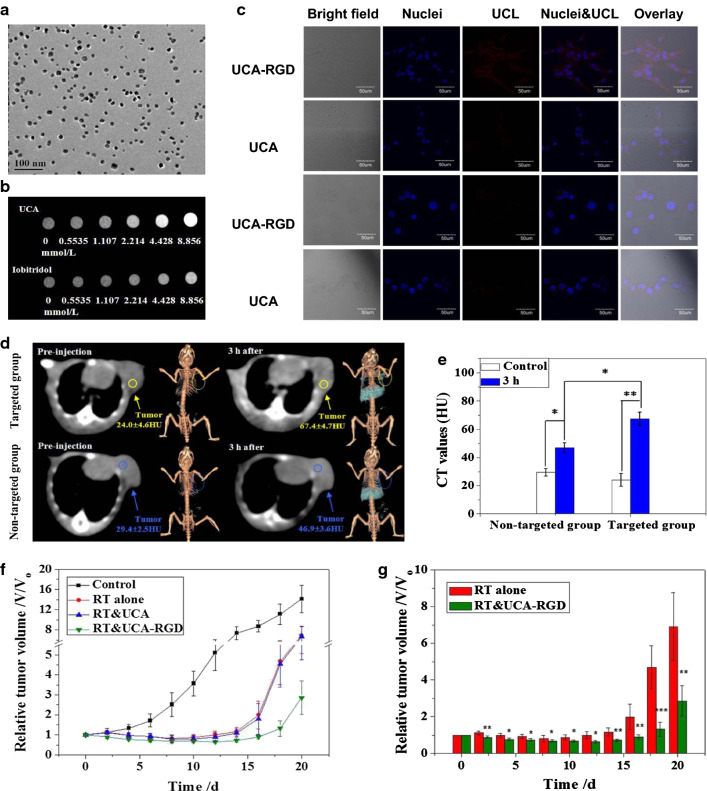
UCA-RGD for CT imaging-guided radiotherapy with effective radiosensitization. **a** TEM characterization of RGD-labelled UCA in water. Scale bar is 100 nm. **b** CT images of UCA and Iobitridol with various concentration. **c** Confocal images of upconversion luminescence (UCL) with UCA-RGD and UCA for 1-h incubation at 800 μg Yb/mL. The two lines above are conducted with U87MG cells and the two lines below are conducted with MCF-7 cells. **d** In vivo transverse slices and 3D volume rendering CT images for U87MG tumor-bearing mice. The comparison includes UCA-RGD (targeted group) or UCA (non-targeted group). **e** CT values of tumor which coincide with (**d**). **f**, **g** Tumor growth inhibition following several types of compare and contrast treatment (Control, RT alone, RT&UCA, RT&UCA-RGB). BaYbF_5_:2% Er^3+^ nanocube (UCA) conjugated with arginine-glycine-aspartic acid (RGD) peptides (UCA-RGD) (Reproduced with permission from reference: [126], copyright 2013 Springer Nature)

Combining multiple elements in high-Z Metal NPs could further enhance the abilities for both imaging and therapy. In 2013, Xing et al. developed upconversion nanocubes made of barium, ytterbium, fluorine, and erbium core conjugated with arginine–glycine–aspartic acid (RGD) peptides which were actively targeted to integrin receptors expressed on tumor neovasculature (Fig. 12a, c). In U87MG tumor-bearing mice, the NPs could be used as a contrast agent for CT and upconversion fluorescence imaging (Fig. 12b–e). In addition, the radiosensitizing effect of the NPs was confirmed in vivo (Fig. 12f, g) [126]. Sun et al. assessed a theranostic ability of the mixed gold and superparamagnetic iron oxide nanoparticle coated with PEG–PCL polymer, an FDA-approved biodegradable co-polymer. In U251 and U373 GBM cell lines, gold and SPION-loaded micelles (GSMs) demonstrated the radiosensitizing effect in vitro and enhanced MRI contrast in heterotopic flank and orthotopic xenograft rodent models [127].

### Strategies to enhance radiosensitizing effects

Crossing the BBB is an important requirement in brain tumor targeting. The BBB is a brain protective system from circulatory system composed of brain endothelial cells and end-feet of astrocytes [128]. The BBB allows passive diffusion of gases and small lipophilic molecules and actively transports nutrients by receptor-mediated endocytosis or absorptive-mediated transcytosis. Although the NPs do not readily pass the BBB, intravenously injected high-Z metal NPs could reach the orthotopic GBM tumors, probably because of the disrupted BBB in tumor models [37, 88, 114, 115, 122, 127]. Many reports suggest the nanoparticles under 100 nm to increase the chance of passive tumor targeting [129]. A shape of gold nanoparticles also affects the in vitro cellular internalization. Spherical gold nanoparticles increased cellular internalization and ROS generation than gold nanorods and gold nanospikes in KB cells, but different shaped NPs showed the same radiosensitization efficiency [130]. In addition, encapsulation with liposomal layers, micelles or PEGylation would be a solution for preventing opsonization and enhanced cell uptake with biocompatible materials. For instance, in vitro cell uptake and radiosensitization effect was enhanced with liposomal encapsulated cisplatin and oxaliplatin compared with liposomal-free platinum compounds in F98 glioma cells [131]. Other researchers reported that chemotherapeutic effect of temozolomide with lipid nanocarriers showed better tumor regression than TMZ solution in subcutaneous glioblastoma models [132]. However, it is still needed to evaluate the tumor delivery efficiency of high-Z metal nanoparticles with different shapes or biocompatible nanocarriers in orthotopic tumor model. In addition, there have been studies on strategies to enhance GBM targeting by antibody conjugation, and prior radiotherapy before NPs administration. For active targeting of glioblastoma, integrins located on brain vascular endothelial cells are the most commonly utilized targets. Active targeting strategies in GBM model include (1) RGD peptides conjugation for the integrin αvβ3 targeting, (2) interleukin 13 peptide conjugation for IL-13Rα2 receptor targeting, and (3) transferrin conjugation for the transferrin receptor targeting [120, 133–135]. Further studies are needed to find an optimal method to improve the efficiency NPs delivery to the brain.

Stimuli-triggered metal nanoparticles also have their potential role in GBM treatment. For example, Shen et al. developed renal-clearable coordination polymer nanodots containing tungsten ions and gallic acid for chelator-free radiolabeling. They successfully labeled radioisotope for tracing the nanoparticles with PET imaging using phenolic hydroxyl group of gallic acid with about 88.34% of labeled ^64^Cu. It was rapidly cleared showing lower retention in liver and spleen at 24 h (13.21%ID/g for liver, 9.01%ID/g for spleen) and 14 d (< 0.2%ID/g for both organs). It was also effective with radiotherapy in 4T1 tumor bearing mice [136]. Another group used liposomal nanoparticles encapsulating gallic acid-ferrous nanoparticles and l-buthionine sulfoximine for synergistic chemo and radio therapeutic effect. Gallic acid-ferrous nanoparticles are efficient in ROS production using Fenton reaction of ferrous iron and ferrous iron oxidation protection of gallic acid. About 5.99% ID/g of the nanoparticles accumulated in tumor at 24 h post injection. The liposomes containing gallic acid-ferrous nanoparticles and L-buthionine sulfoxamine efficiently suppressed cancer growth than a group treated free L-buthionine and gallic acid-ferrous nanoparticles [137]. Photo-induced synergistic cancer therapy is emerging as a new strategies of cancer therapy. Zhong et al. developed PEGylated NaCeF_4_:Gd, Tb nanoparticles for multimodal imaging and radiotherapy. Ce and Tb ions produce ROS by absorbing the energy of X-ray. Also, lanthanide elements act as a radiosensitizer and CT imaging contrast agent. Researchers allowed of multimodal X-ray fluorescence, CT and MR imaging by the nanoparticles. Also their in vivo radiosensitization and radiodynamic therapy efficiency were better than X-ray radiation only in A549 mouse tumor model [138]. For NIR light induced photothermal therapy and radiosensitization, authors developed liposomal nanoparticles encapsulating iridium nanocrystals. The NIR light irradiated nanoparticles enhance tumor oxygenation by catalyzing H_2_O_2_ in tumor. Also, iridium elements worked as a radiosensitizer and contrast agent in photoacoustic imaging [139].

## Conclusion and future perspectives

This review described the mechanisms of radiosensitization by high-Z metal NPs and summarized the literature describing high-Z metal NPs mediated radiosensitization in GBM. The mechanism of radiosensitization by high-Z metal NPs is not confined to the physical aspect alone but extended to chemical/biological effects. Multiple in vitro and pre-clinical in vivo studies proved the radiosensitizing effect of high-Z metal NPs in different GBM models. Although various types of high-Z metal NPs were found to be useful for radiosensitization, there have not been enough studies to systematically compare the efficacy of different types of NPs, except for one study that reported the superiority of the silver NPs over gold NPs [115]. Targeting efficiency of high-Z metal NPs could determine the radiosensitizing efficiency because specific accumulation of the NPs in the tumor cells enhance the specific radiosensitization of cancer tissue compare to surrounding normal tissue. The strategies for crossing the BBB and active targeting would enhance the targeting ability of the NPs. Furthermore, intracellular location of the NPs affects the radiosensitizing effect. Thus, it is recommended to develop (1) intracellular targeting strategies of NPs, desirably to nucleus or mitochondria, along with (2) micro dosimetry methods to monitor the different radiosensitizing effect in micro level. Also, further studies are warranted to elucidate factors that can be used to maximize the radiosensitizing effect by comparing different types of the metal elements, functionality, size, and shape.

The renal and hepatic pathways are the common nanoparticle excretion pathways to reduce the toxicity. However, inorganic nanoparticles such as gold NPs and iron oxide NPs are often confined in the liver and spleen by reticular endothelial system than renal clearance. The particle size decrease and surface functionalization are a key factor of designing renal clearable and long circulating nanoparticle for avoiding the toxicity with rapid elimination and increased targeting efficiency [140]. Only a few reports investigated about neurotoxicity of metal nanoparticles. The authors compared silver, copper and aluminum nanoparticles in the same size (50–60 nm) with different administration methods. Ag and Cu nanoparticles have disrupted the BBB permeable function than Al nanoparticles showing their brain edema and cell damage [141, 142]. However, not only are there very few papers on brain toxicity of nanoparticles, but in this paper, Ag nanoparticles are injected more than the other references about radiosensitizer of high-Z nanoparticles in Table 1. Thus, we need systemic studies on brain dysfunction of high-Z nanoparticles to find the optimal dose to show the therapeutic effect as long as there is no toxicity. Otherwise, intratumoral injection of NPs may be a realistic option for a faster clinical translation. Further, to date, not enough in vivo studies have evaluated toxicity, biodistribution, and excretion of the radiosensitizing high-Z metal NPs. In this respect, more investigations are required to assess the behavior of NPs in vivo to facilitate the clinical translation of this promising therapeutic strategy for treatment of GBM.

## Data Availability

Not applicable.

## Abbreviations

GBM: Glioblastoma multiforme
NPs: Nanoparticles
EPR: Enhanced permeability and retention
BTSG: Brain Tumor Study Group
WBRT: Whole-brain radiotherapy
IFRT: Involved-field radiotherapy
SRS: Stereotactic radiosurgery
IMRT: Intensity-modulated radiotherapy
EGFR: Epidermal growth factor receptor
LET: Linear energy transfer
DEF: Dose enhancement factor
ROS: Reactive oxygen species
DSB: Double-strand break
BBB: Blood–brain barrier
SER: Sensitizer enhancement ratio
MGd: Motexafin gadolinium
AGuIX: Activation and Guidance of Irradiation by X-ray
HUVEC: Human umbilical vein endothelial cells
SPIONs: Superparamagnetic iron oxide nanoparticles
